# “It depends how one understands it:” a qualitative study on differential uptake of oral cholera vaccine in three compounds in Lusaka, Zambia

**DOI:** 10.1186/s12879-019-4072-6

**Published:** 2019-05-14

**Authors:** Leonard W. Heyerdahl, Miguel Pugliese-Garcia, Sharon Nkwemu, Taniya Tembo, Chanda Mwamba, Rachel Demolis, Roma Chilengi, Bradford D. Gessner, Elise Guillermet, Anjali Sharma

**Affiliations:** 1 Agence de Médecine Préventive, J 87, Deux Plateaux, Abidjan, Côte d’Ivoire; 2 École normale supérieure de Lyon, UMR 5206 Triangle, Lyon, France; 3 Centre for Infectious Disease Research in Zambia (CIDRZ), Plot # 34620, P.O. Box 34681, 10101 Lusaka, Zambia; 4 Agence de Médecine Préventive, 13 chemin du Levant, 01210 Ferney-Voltaire, France

**Keywords:** Cholera, Belief, Vaccine hesitancy, Zambia

## Abstract

**Background:**

The Zambian Ministry of Health implemented a reactive one-dose Oral Cholera Vaccine (OCV) campaign in April 2016 in three Lusaka compounds, followed by a pre-emptive second-round in December. Understanding uptake of this first-ever two-dose OCV campaign is critical to design effective OCV campaigns and for delivery of oral vaccines in the country and the region.

**Methods:**

We conducted 12 Focus Group Discussions (FGDs) with men and women who self-reported taking no OCV doses and six with those self-reporting taking both doses. Simple descriptive analysis was conducted on socio-demographic and cholera-related data collected using a short questionnaire. We analyzed transcribed FGDs using the framework of dose, gender and geographic location.

**Results:**

No differences were found by gender and location. All participants thought cholera to be severe and the reactive OCV campaign as relevant if efficacious. Most reported not receiving information on OCV side-effects and duration of protection. Those who took both doses listed more risk factors (including ‘wind’) and felt personally susceptible to cholera and protected by OCV. Some described OCV side-effects, mostly diarrhoea, vomiting and dizziness, as the expulsion of causative agents. Those who did not take OCV felt protected by their good personal hygiene practices or, thought of themselves and OCV as powerless against the multiple causes of cholera including poor living conditions, water, wind, and curse. Most of those who did not take OCV feared side-effects reported by others. Some interpreted side-effects as ‘western’ malevolence. Though > 80% discussants reported not knowing duration of protection, some who did not vaccinate, suggested that rather than rely on OCV which could lose potency, collective action should be taken to change the physical and economic environment to prevent cholera.

**Conclusions:**

Due to incomplete information, individual decision-making was complex, rooted in theories of disease causation, perceived susceptibility, circulating narratives, colonial past, and observable outcomes of vaccination. To increase coverage, future OCV campaigns may benefit from better communication on eligibility and susceptibility, expected side effects, mechanism of action, and duration of protection. Governmental improvements in the physical and economic environment may increase confidence in OCV and other public health interventions among residents in Lusaka compounds.

## Background

Cholera manifests as an acute diarrhoeal infection which, if untreated, can be fatal for up to half the affected individuals due to rapid dehydration [1, 2]. Ingesting food and water contaminated by *Vibrio cholera* living in water bodies or transmitted through the oral-fecal route, may lead to cholera [2–4]. The World Health Organization (WHO) recommends that low-income countries with endemic and episodic cholera should use a two-dose oral cholera vaccine (OCV), administered 2 to 4 weeks apart, as a preventive strategy in cholera hotspots and as a reactive strategy during cholera outbreaks [5, 6]. This recommendation particularly applies to countries that cannot consistently assure safe drinking water, sanitation, and hygiene (WASH) [2].

The “last-mile problem” is manifest in Zambia’s capital, Lusaka. Persons living in overcrowded informal settlements (also known as “compounds”) are unwilling to pay for safe water and sewage and the government is unable to subsidize households’ connections to existing water and sewage networks [7]. Hence, inhabitants acquire their drinking water from shallow wells and kiosks. Water for domestic use usually comes from shallow wells located within 5–10 m of pit latrines [8, 9]. Pit-latrines are rarely emptied and rest on rocky terrain, which if flat become a natural drainage plain for the city [10]. Flooding during the rainy season carries contaminants into ground water from overflowing pit-latrines, as well as from “flying toilets” (cardboard containers of feces thrown away from the home), open defecation, and uncollected garbage [9, 10]. Such an environment, accompanied by rural-urban travel and poor food and personal hygiene, is conducive to cholera outbreaks in Lusaka compounds [8, 9].

From February to May 2016, cholera broke out in seven Lusaka compounds with affected clinics reporting 1054 cases and 20 deaths [8]. The outbreak began in Kanyama compound and within a month had spread to compounds in Chawama and Bauleni, spanning an area of 12–15 km [8]. To stop further transmission, the Zambian Ministry of Health (MoH) and Médecins Sans Frontières (MSF) added a reactive one-dose OCV campaign to standard control measures in these three compounds [9]. Shanchol, an inactivated OCV, was chosen for its convenience (since it requires no buffer) [11], safety [12], cumulative efficacy of up to 65% for < 5 years [13], and proven success in reactive OCV campaigns conducted in Africa [14]. The one-dose OCV campaigns were selected based on modelling suggesting that administering all the available OCV using one-dose instead of two to vaccinate more people during a cholera outbreak may avert more cases and deaths [15]. Also, a randomized controlled study from Bangladesh testing single-dose Shanchol, albeit not in outbreak settings, had demonstrated vaccine efficacy of 40% at 6 months and 63% against severely dehydrating cholera [13]. From 9 to 25 April 2016, one dose of Shanchol was delivered to 423,774 people more than 1-year of age living in Chawama, Kanyama and Bauleni compounds (≈78% of the target population) [11]. The MoH with support from the WHO Zambia office administered a second dose of Shanchol in December 2016 to 437,140 people more than 1-year of age in the three compounds.

Understanding factors influencing participation in the two OCV campaigns in Lusaka can help the MoH better prepare and execute future OCV campaigns. Studies from other countries show that perceptions of cholera, past experience with vaccinations, social disenfranchisement/marginalization, fear of vaccine side effects, and opportunity costs deter participation in OCV campaigns [16–22]. In this paper, we compare the perspectives of those who self-reported 1) not receiving the vaccine (non-vaccinated) and 2) receiving both OCV doses (fully vaccinated) in the three compounds to understand factors that could impact this population’s participation in future campaigns in Zambia. This study adds to the limited literature on contextualized assessments to anticipate and better understand vaccine uptake challenges undertaken in Africa for OCV and oral vaccines in general [23–26].

## Methods

This analysis is nested in a larger rapid qualitative assessment conducted before, during, and after the second-dose OCV campaign carried out in December 2016 in the three compounds to understand community and health worker perspectives and experience through 48 Focus Group Discussions (FGDs) and 25 Key Informant Interviews (KII). In this analysis, we compare responses from 18 Focus Group Discussions (FGDs) conducted in the three compounds, including six each with men and women who reported being unvaccinated at the first and second campaigns respectively (henceforth referred to as non-vaccinated) and six with men and women who reported being fully vaccinated men and women by the end of the second campaign (referred to as fully vaccinated from hereon). While the larger study provides the rich context around people’s perspectives on cholera, vaccines, OCV and the OCV campaigns, this sub-analysis, based on people’s dose-specific experience, allows a more granular description of the factors that influenced uptake of OCV. Given the sheer amount of available data and as we aimed to have a clear division between non-vaccinated and fully vaccinated participants, FGDs with participants who had one dose (partially vaccinated) were not included in this analysis. The authors aim to publish findings from among partially vaccinated FGD participants in a separate publication.

### Sample size considerations

Recent empirical work supports the long held view that one to three FGDs with a single type of discussant reaches at least 80% saturation of information and three to six FGDs more than 90% [27]. In this paper, we draw on 18 FGDs: six FGDs each for men and women who self-report not getting OCV during the first and second campaigns, as well as three FGDs each for fully vaccinated men and women for a total of 171 participants (see Table 1).

**Table 1:** Key socio-economic characteristics in the focus group discussions respondents

Characteristics	0 doses *n* (%)	2 doses *n* (%)	Total *N* (%)
	*N* = 113	*N* = 58	*N* = 171
Gender			
Female	58 (51)	31 (53)	89 (52)
Male	55 (49)	27 (47)	82 (48)
Didn’t answer	0 (0)	0 (0)	1 (1)
Age range			
18–20	15 (13)	9 (16)	24 (14)
20–35	76 (67)	39 (67)	115 (67)
36–50	13 (12)	8 (14)	21 (12)
51–68	7 (6)	2 (3)	9 (5)
Didn’t answer	2 (2)	0 (0)	6 (4)
Compound			
Bauleni	36 (32)	18 (31)	54 (32)
Chawama	38 (34)	23 (40)	61 (36)
Kanyama	39 (35)	17 (29)	56 (33)
Education			
Below High School	43 (38)	24 (41)	67 (39)
High School	49 (43)	29 (50)	78 (46)
Above High School	20 (18)	5 (9)	25 (15)
Didn’t answer	1 (1)	0 (0)	(0)
Religion			
Christian	110 (97)	58 (100)	168 (98)
Other	0 (0)	0 (0)	0 (0)
Didn’t answer	3 (3)		
Has children			
No	47 (42)	22 (38)	69 (40)
Yes	66 (58)	36 (62)	102 (60)
Agrees to give Oral Cholera Vaccine to their children (% with children who agree)			
No	60 (91)	0 (0)	60 (59)
Yes	5 (8)	36 (100)	41 (40)
Don’t know	1 (2)	0 (0)	1 (1)
Reported diseases in the community			
Malaria	89 (79)	56 (97)	145 (85)
Diabetes	17 (15)	26 (45)	43 (25)
Cholera	94 (83)	48 (83)	142 (83)
Polio	19 (17)	11 (19)	30 (18)
Tuberculosis	50 (44)	42 (72)	92 (54)
HIV/AIDS	12 (11)	14 (24)	26 (15)
Other	5 (4)	8 (14)	13 (8)
Total	113	58	171

### Sampling

We used convenience sampling to recruit discussants. Two male and two female research assistants walked daily from a different 2016 OCV delivery post in each compound to the nearest adult gathering (for example, the market place) to identify those living in cholera hotspots. They provided a brief explanation of the study to individuals before inviting participation. They continued recruiting at the house closest to the gathering, moving in concentric circles until they reached the required sample size (8–12 adults) per FGD. Willing adults were given an appointment to attend a FGD at the health care clinic serving the compound. Appointments were specific to gender and self-reported cholera vaccination status – zero or one – before the second-dose campaign. During and after the second-dose campaign, those who had received two doses were also invited to participate in FGDs. Names and phone numbers of those who agreed to attend were noted. No-shows to the scheduled FGDs were contacted by phone. Persons who could not attend were immediately replaced by new recruits within the vicinity of the health clinic.

### Enrolment and data collection

All participants received information on the study in their chosen language (Bemba, English, Nyanja). After confirming comprehension, they gave written informed consent to voluntary study participation and audio-recording. No recruit who came to the clinic refused or withdrew from the FGDs.

At FGDs, discussants filled in a short questionnaire on their socio-demographic characteristics and on their perceptions of disease and cholera facilitated by Research Assistants. The FGD guide included a set of predetermined questions covering topics on perception of cholera causation, vaccines, and OCV acceptability; practices to prevent and treat cholera; and logistical inputs on space and timeline of vaccine delivery. The FGD Guide was designed on the basis of qualitative assessments of OCV campaigns in Malawi and Mozambique conducted by two authors of this paper [28, 29]. One Research Assistant facilitated the FGD while another recorded and took notes. Participants identified themselves by token numbers to maintain confidentiality and for ease of analysis. They were reminded not to share information discussed during FGDs. FGDs lasted 1.5 to 3 h. FGD participants were offered refreshments and compensated with 20 Zambian Kwacha (ZMW; ~ US$2) for travel.

### Data management and analysis

Field notes and English transcriptions of interviews held in Bemba or Nyanja were entered in Microsoft Word© and short questionnaires in Microsoft Excel©, all of which were uploaded to Nvivo©. Transcripts were checked against approximately half the FGD recordings. Finding no major problems, no further checks were conducted.

Socio-demographic characteristics and knowledge and perceptions of cholera were analyzed using simple frequencies and percentages. In a first cycle of coding, transcripts were coded using pre-determined codes derived from the FGD guide. Based on emerging themes and analytical connections, a second cycle of coding using the HBM constructs (perceived susceptibility, severity of the disease, benefits from vaccinating with OCV, barriers to vaccination) was applied. Coders maintained a coding journal and a memo repository on codes, sub-codes, and analytical connections. This reflexive data processing allowed creating or re-adapting codes when coders decided this was needed. Data were stratified by participants’ residential location, OCV dose uptake and gender before being exported as Microsoft Word© tables for synthesis and analysis. Framework analysis was used to understand differences in responses.

The perspectives of those non-vaccinated and those fully vaccinated during the first (April 2016) and second dose campaigns (December 2016) are presented below. We frame these sub-sections by the constructs offered by the health belief model as they best fit the themes emerging from our analysis on the phenomena of full OCV uptake or none at all [30, 31]. The health belief model holds that the adoption of prevention measures depends on whether people believe that they are vulnerable to the disease (perceived susceptibility); that the disease comes with severe health or social consequences (perceived severity); and that the benefits of preventing the disease (perceived benefits) outweigh the barriers of adoption such as cost, time, pain, and side-effects (perceived barriers) [30]. In addition, we present the influence of cultural beliefs regarding disease causation and social interactions on OCV uptake [32]. A summary table of the HBM structured thematic coding can be found in Appendix.

## Results

### Participant characteristics

Each FGD had between 8 and 12 participants for a total of 171 discussants, of whom 89 were female and 82 were male. Male and female participants were equally spread across the three compounds, 98% declared they were Christian, 81% were 18–35 years old and 39% had below high school education (see Table 1). In total, 60% of participants in each group had children and 91% of those in the non-vaccinated group said they would not give their children OCV. Both groups listed the following diseases experienced in their community in decreasing order of frequency: malaria (85%), cholera (83%), tuberculosis (54%), diabetes (25%), and polio (18%) HIV/AIDS (15%).

### Disease recognition, causation and perceived susceptibility

In the short survey, 71% identified vomiting as a symptom of cholera. Fewer (40% non- and 57% fully vaccinated) identified diarrhoea as a symptom of cholera (see Table 2).

**Table 2:** FGDs participants’ knowledge and preferences on key aspects around cholera expressed as number and percentage of respondents by oral cholera vaccine dose (multiple answers allowed)

Characteristics	0 doses *n* (%)	2 doses *n* (%)	Total *N* (%)
	*N* = 113	*N* = 58	*N* = 171
Known cholera symptoms			
Fever	3 (3)	0 (0)	3 (2)
Diarrhoea with blood	4 (4)	0 (0)	4 (2)
Diarrhoea without blood	45 (40)	33 (57)	78 (46)
Vomiting	80 (71)	41 (71)	121 (71)
How can you get cholera			
Mosquito	0 (0)	0 (0)	0 (0)
Dirtiness	82 (73)	40 (69)	122 (71)
Wind	17 (15)	16 (28)	33 (19)
Contact with dead body	20 (18)	18 (31)	38 (22)
Dirty water	60 (53)	40 (69)	100 (58)
Dirty food	63 (56)	42 (72)	105 (61)
Flies	60 (53)	37 (64)	97 (57)
Curse	4 (4)	0 (0)	4 (2)
God’s punishment	0 (0)	0 (0)	0 (0)
How can you treat cholera			
Water with salt	5 (4)	1 (2)	6 (4)
Traditional healer	3 (3)	2 (3)	5 (3)
Oral rehydration salts	55 (49)	23 (40)	78 (46)
Hospital	91 (81)	45 (78)	136 (80)
At home	1 (1)	3 (5)	4 (2)
Street antibiotics	0 (0)	0 (0)	0 (0)
Drugstore	6 (5)	7 (12)	13 (8)
Prayer/pastor	2 (2)	4 (7)	6 (4)
Total	113	58	171

To a greater extent, those fully vaccinated attributed cholera to dirty water (69%) and dirty food (72%) as compared to the non-vaccinated participants (53 and 56%, respectively). Four of the non-vaccinated participants believed that cholera appeared as a consequence of a curse, whereas none of the fully vaccinated participants did.

More respondents in the fully vaccinated group believed that cholera may be contracted following contact with a dead body (31% vs. 18%) and that flies were contaminating agents (64% vs 53%).

Lastly, 28% of fully vaccinated participants and 15% of non-vaccinated participants believed cholera to be an airborne disease. In the FGDs, the fully vaccinated stated that cholera was both waterborne and airborne against which OCV was necessary protection. Those who were not vaccinated reported that the protection offered by OCV against an airborne disease was inadequate. The quotes below illustrate these perspectives:

P8: *“I did not have interest in it [OCV] because I believed that cholera can move through water and by air … So if I am meant to get sick, then I will! That is why I did not even go.”*

**An unvaccinated man from compound B, FGD conducted after the campaign**

P1: *“Others have said that it [cholera transmission] is by air. It’s better that you go and drink the medicine [OCV]. Even if the medicine is bad, it does protect. You just need to drink it.”*

**A woman who received two doses from compound B, FGD conducted after the campaign**

As shown in Table 2, non-vaccinated participants were slightly more prone to declaring treating cholera at the hospital (81%) or with rehydration salts (49%), compared to fully vaccinated participants (78 and 40% respectively).

All participants irrespective of their vaccination status thought that other preventive measures such as hand washing or treating water should be maintained. At least 53% of the non-vaccinated and 33% of the vaccinated (see Table 3) thought they would be less careful about personal hygiene if vaccinated. Both groups (72% non-vaccinated and 65% fully vaccinated) anticipated that those who relied on the vaccine could forget about hygiene. Several unvaccinated participants thought that this neglect of WASH put those vaccinated at higher risk of contracting cholera when the vaccine lost potency:

P3: “*If one wants to drink [OCV], you can’t stop them! But, whichever way, I believe it all comes back to personal hygiene; how you take care of yourself, your surroundings, how you keep your surroundings. Because that’s [OCV] just a prevention. It [OCV] is not a treatment for a very long time. Eventually it will be out of the body, so if it is out of the body before the cholera thing is cleared in the community, one can get cholera … You need to maintain good personal hygiene. I think that’s the best way of protecting yourself.”*

**An unvaccinated woman from compound C , FGD conducted before the campaign**

One participant explained the difference between prevention and protection and described prevention as being less reliable as follows:

P2: “*If I protect something, it means, there is nothing that can penetrate it because that thing is protected. When we talk about prevention, I think it only applies to certain measures, only at a certain level, to prevent something from entering. Like for example, if someone takes the vaccines, I don’t think you can say that they are protected 100% from cholera! No!”*

**An unvaccinated man from C compound, FGD conducted after the campaign**

**Table 3:** Duration of oral cholera vaccine protection and impact of the vaccine on other preventive practices

Characteristics	0 doses *n* (%)	2 doses *n* (%)	Total *N* (%)
	*N* = 113	*N* = 58	*N* = 171
How long does 1 dose of the oral cholera vaccine last			
Six months	7 (6)	5 (9)	12 (7)
One to five years	0 (0)	1 (2)	1 (1)
Lifetime	15 (13)	0 (0)	15 (9)
Don’t know	69 (61)	35 (60)	104 (61)
Didn’t answer	22 (19)	17 (29)	39 (23)
How long do 2 doses of the oral cholera vaccine last			
Six months	4 (4)	2 (3)	6 (4)
One to four years	4 (4)	5 (9)	9 (5)
Five years	3 (3)	3 (5)	6 (4)
Lifetime	5 (4)	0 (0)	5 (3)
Don’t know	74 (65)	25 (43)	99 (58)
Didn’t answer	23 (20)	23 (40)	46 (27)
Believe vaccination will change their own hand washing practices or seeking improved water quality			
No	28 (25)	31 (53)	59 (35)
Yes	60 (53)	19 (33)	79 (46)
Maybe	0 (0)	0 (0)	0 (0)
Don’t know	3 (3)	0 (0)	3 (2)
Didn’t answer	22 (19)	8 (14)	30 (18)
Believe others will be less careful with water and sanitation			
No	16 (14)	8 (14)	24 (14)
Yes	74 (65)	42 (72)	116 (68)
Maybe	4 (4)	0 (0)	4 (2)
Don’t know	0 (0)	0 (0)	0 (0)
Didn’t answer	19 (17)	8 (14)	27 (16)
Total	113 (66)	58 (34)	171

Several discussants who were not vaccinated did not feel personally susceptible to cholera due to their hygiene practices, with one man in Bauleni explaining that, *“Me, I can’t get sick because I know how I take care of myself!”.* Others observed that the vaccine appeared to be for children, saying that, *“because only kids are the ones that I saw going there* [to get the vaccine]” with some clarifying, “*mostly because they are at higher risk of getting sick. They play too much*.” Participants from both the non-vaccinated and fully vaccinated groups declared that children and the poor were more vulnerable.

Both those in the fully vaccinated and non-vaccinated groups were knowledgeable about the severe consequences of cholera. However, those who had taken both doses of OCV appeared to have a heightened awareness of the life-threatening nature of cholera, mentioning its visible impact on the body and how it depleted bodily fluids quickly and could kill within “1–24” hours. Two FGDs of fully vaccinated discussants had at least one participant who stated that: i) not everyone thought of cholera as serious, otherwise everyone would get vaccinated; and ii) people only took cholera seriously if they saw it attack someone. While at least one member from each of the fully vaccinated FGDs reported knowing someone that they believed had suffered cholera illness during the outbreak, 10 of 12 non-vaccinated FGDs also reported the same, suggesting that such knowledge is insufficient to motivate OCV uptake.

### Perceived benefit of getting OCV

During discussions, a majority of discussants noted that cholera cases seemed to have reduced since OCV introduction. For this reason, even among those not vaccinated, some presumed that OCV may be effective. Conversely, the absence of cholera after the reactive one-dose campaign in April 2016, convinced those who took the first dose to either intend or actually take the second dose. This was as narrated by four members of a FGD after the campaign:

P6: *“Why we have liked the vaccines is because it prevents us from the disease. This year, there is no [treatment] tent for cholera.”*

**A woman who received two doses from compound K, FGD conducted after the campaign**

Those not vaccinated expressed doubts about vaccine efficacy, saying that, “*I did not believe that it will work for five years. That’s why I did not go there* [to take OCV].”. One non-vaccinated man in Bauleni emphasized that “*We don’t know the statistics, so we can’t say how safe they [OCVs] are. Because people are just taking vaccine and we have not seen the result.”*

As Table 3 illustrates, > 80% of discussants in both groups did not know or did not respond when asked the duration of protection offered by one-dose and two doses of OCV in the short survey. With that said, in the FGDs, most fully vaccinated participants demonstrated an understanding of the limited period of OCV efficacy. Five of the six FGDs whose members were fully vaccinated volunteered information on the protective period ranging from 6 months for one dose and 2–3 years for the second dose with some specifying that it did not mean that campaigns would be every 3 years. Two people categorically stated nothing was mentioned regarding protection conferred by one dose. Some participants drew on their experiences of medication used for other ailments to justify a similar need for the second dose:

P8: *“For me, it was like when I picked the first dose, the second dose was important because it was completing the medicine and the medicine would work effectively. Like medicine for T.B., if you don’t complete the dose, you stop [the treatment], you can fall sick.”*

**A man who received two doses, from K compound, FGD conducted after the campaign**

For the non-vaccinated participants, the benefits for adults was not so clear, particularly given the unknown side-effects and the possibility of ignoring other protective practices:

P4: *“Us, we don’t know because we have not drunk [OCV]. So we don’t know whether they can prevent us [from getting cholera] or what?! Because we are scared of these reactions that react on our bodies when we take such vaccines.”*

**An unvaccinated man_ from K compound, FGD conducted after the campaign**

P0: “*This medicine does not assure me that I will not get sick. So they are not 100% ... When you drink [OCV], you need to protect yourself. Then you can be assured that you will not get sick. But if you do not protect yourself because you have drunk the medicine then you, surely, you will be affected*.”

**An unvaccinated man_ from C compound, FGD conducted after the campaign**

As suggested by the sub-section on perceived susceptibility to cholera, a few non-vaccinated discussants thought that OCV could however protect children who were more vulnerable and less able to take care of themselves. This is concordant with the 8% of non-vaccinated who said they would accept OCV for their children in the short survey (See Table 1).

### Perceived barriers to OCV uptake

Access to information during the campaign, cultural understanding of medicinal potency and perceived side-effects influenced OCV uptake. The main deterrent to taking OCV was fear of side-effects which were reportedly passed on from those who had been vaccinated rather than from reliable professional sources. Those vaccinated reported developing a rash (*vipeele)*, experiencing a drunkenness sensation, insomnia, loss of appetite, stomach ache, dizziness, nausea, diarrhoea and vomiting. Other mild side-effects reported included feelings of severe weakness, nausea, vomiting, “small diarrhoea,” temporary stomach pains and a rash.

Many among those non-vaccinated were persuaded not to take OCV due to these reported side-effects:

P3: “*The way everyone is saying that each one had experienced some side effect like diarrhoea for three days … Then me, the way I am, I have that type of stomach; with diarrhoea with vomiting, I am usually faster [very susceptible]*.”

**An unvaccinated woman from C compound, FGD conducted after the campaign**

Those non-vaccinated expressed concerns such as: “*If I drink, I will be sick*” and that OCV may “*cause long-term side-effects*.” Some warned that if there are coincidental illnesses and death, “*people will attribute that to the cholera vaccine,*” a point illustrated by the quote below:

P4: “*With me, I saw my sister who had just come from taking the vaccine. So I found her … looking like she wanted to vomit. Then I asked her, “What is it?” Shortly after asking her, “What is it?,” she held her stomach, then rushed to the toilet. Then I said, “Aah! No! You can be sick too!” She had diarrhoea for three days. I don’t know whether she just had diarrhoea or it’s because of the vaccine. So that’s how I also got scared that I can also have diarrhoea*.”

**An unvaccinated woman from C compound, FGD conducted after the campaign**

The taste of the vaccine also influenced uptake. Those vaccinated described the taste of OCV as “bad,” “bitter,” “funny,” “like rotten eggs,” “salty,” “chlorine,” “raw grasshoppers,” and “raw eggs.” While non-vaccinated said that these reports dissuaded them, several of fully vaccinated coped by downplaying the taste. One man stated the bad taste was “*only 15 minutes*”; some that they had become accustomed to the taste by the second dose; and, others that they were given water or bought mints and sweets to get rid of a taste that would otherwise last in one’s mouth. For instance, one woman shared that:

“*The medicine, it tasted like rotten eggs; so I felt nausea. I felt nausea till I bought some chewing gums.”*

**A woman who received two doses from compound K, FGD conducted after the campaign**

Some suggested that a larger vial would make it easier to sip in one go instead of having to take several sips to ensure that they drained the current “*small bottle*.”

Cultural notions regarding medicines influenced uptake. A few among those non vaccinated thought of “*medicine*” as “*strong*” and disrupting to the body. In addition, they thought that blood needed to be strong to withstand the assault of the vaccine as illustrated by the quotes below. This might explain, in part, participants’ dislike and reluctance to take medication.

P3: *“You find that the medicine is very strong. So if your blood is weak and you go and get the medicine, it can affect other things! So what they do, they go and get the medicine. After taking, then they collapse! So those are the impact [effect] that prevent people from taking the drugs. They think that if I take maybe I can collapse or something will happen to me’.”*

**An unvaccinated man, FGD from compound B, FGD conducted before the campaign**

For those who were vaccinated, it was normal for a medicine to taste bitter and that side effects simply demonstrated that OCV would prevent cholera:

“*I should just say every medicine has a smell or is bitter. Fansidar [pyrimethamine and sulfadoxine] is bitter and when they drink it, they vomit. That’s how they made the medicine so that it should be preventive.”*

**A woman who received two doses from compound K, FGD conducted after the campaign**

“*There were different reactions with the vaccines. Others had diarrhoea, vomiting and other different side effects like stomach pains. But even though there were those side effect, they were making the body not be spoilt*.”

**A man who received two doses from compound K, FGD conducted after the campaign**

Viewing the side effects as normal led to acceptance of new discomforts. For instance, one discussant who had never had diarrhoea before thought that it was the result of OCV “*just cleaning the stomach.*” Another noted a more prolonged episode, but accepted the side-effects as normal and resisted contrary rumors that were circulating in the compound:

P8: “*It is true, from the time I took the vaccine I had diarrhoea for a week … People in the compound even said, “The white people are the one making cholera” and they are discouraging people. But after the diarrhoea I was fit again, I felt better*.”

**A man who received two doses from compound C, FGD conducted during the campaign**

### Access to information

Most of the participants said that they did not access the vaccine because they were not well informed about who was meant to be vaccinated, the level or duration of protection and the potential side effects, leading to the suggestion that:

P1: *“Maybe before they start to vaccinate people, maybe they should be teaching them so that they have clear information. So, we know that what we are drinking, why we are drinking it: it is because of this and that reason. And then if there is any side effect, they [we] should know that. When we drink this medicine, it will have this and that side effect. Maybe it can be of help to the people who have not drunk yet because the way those who already drank are discouraging – “No! They [OCV] are not nice!”, “You get drunk”, “They [OCV] do this”, “Me! I had rash!” -- Maybe that rash [is because] she just ate something, then she is saying, “it’s the medicine”, So, it is better that you teach them. They even have clear information, to say this medicine you are drinking is like this and that. When you see this sign, then [it means that] the medicine is working. Others with good blood will not have any side effect.”*

**An unvaccinated woman from compound C, FGD conducted after the campaign**

The lack of information regarding OCV, including potential side effects, made the observed side effects all the more disturbing and granted credibility to those participants who warned about the vaccine’s lack of safety, even if based on unfounded rumors. As shown in Table 4, *family and friends advising against OCV* was reported by 63% of non-vaccinated and 26% of fully vaccinated participants as a reason why people may refuse OCV in the survey. In FGDs, one man explained:

*P2: “Even when they heard about the issues to do with cholera, they were scared to drink [OCV] thinking that they can cause reactions just the way measles reacted … Even if I am fine now, maybe by the time I will take the vaccine, I will be drunk. I have already heard from someone saying that even if they [outsiders] make some medicines, they will come and try it on us [to see] if it is working. …. Then when you drink, you will have diarrhoea or you have rash or something. They should just say the name of the vaccine to say there will be this type of the vaccine that you will need to drink is for such type of disease. Now how can you take that if you have not known [what type of vaccine for what disease]?! So you just need people to educate us.”*

**An unvaccinated man from K compound, FGD conducted after the campaign**

**Table 4:** Participants” preferences regarding oral cholera vaccine expressed as number and percentage of respondents by vaccine dose

Characteristics	0 doses *n* (%)	2 doses *n* (%)	Total *N* (%)
	*N* = 113	*N* = 58	*N* = 171
Reasons to refuse oral cholera vaccine (Multiple responses allowed)			
No need	11 (10)	8 (14)	19 (11)
OCV does not work	14 (12)	4 (7)	18 (11)
Family/friend advised against	71 (63)	15 (26)	86 (50)
I prefer injections	14 (12)	11 (19)	25 (15)
Bad experience with vaccine	38 (34)	17 (29)	55 (32)
Preferred days to organize a campaign (Multiple responses allowed)			
Monday	5 (4)	15 (26)	20 (12)
Tuesday	1 (1)	3 (5)	4 (2)
Wednesday	3 (3)	3 (5)	6 (4)
Thursday	0 (0)	15 (26)	15 (9)
Friday	13 (12)	3 (5)	16 (9)
Saturday	60 (53)	32 (55)	92 (54)
Sunday	46 (41)	22 (38)	68 (40)
Prefer fixed or mobile campaign			
Fixed	26 (23)	9 (16)	35 (20)
Mobile	78 (69)	33 (57)	111 (65)
Total	113	58	171

Some respondents related rumors of the vaccine being a mischievous tool deployed by “whites”:

P0: “*Some people say it’s a way that whites want to kill us through the vaccine by bringing something that we do not really understand … When someone says something, the first thing, it will just stick to your head. When someone just comes and say- “The injection that you are getting, that’s how whites kill you, because the white complain that today cholera, today cholera.” - These are the people that discourage us*.”

**An unvaccinated woman from compound C, FGD conducted after the campaign**

Discouragement from those who were vaccinated and lack of accurate information to counteract rumors, especially in poor areas, exacerbated the fear of side-effects:

P6: *“I think discouragement goes together with lack of information, advertising and the place. So you guys have not gone to advertise in low density areas. It’s all about the high density.”*

**An unvaccinated man from compound C, FGD conducted after the campaign**

Most of those who were vaccinated heard about the campaign from community sources such as “people from health going around campaigning” and announcing it over a megaphone, community sensitization in school, church, home visits, including from a “little boy,” clinic, friends, and family (including those working in health). Only one person mentioned radio and two cited television as sources of information. Most people opted for both OCV doses because they believed their sources when they said that they should protect themselves, preserve their health, and preserve their lives through OCV:

“O*thers were saying, the taste [of OCV] is bitter but let’s just go and drink because we are protecting ourselves, it does not look good when you die because they are going to bury you like a dog, it's better you prevent yourself. Others were drinking, very few were refusing.*”

**A woman who received two doses from compound K, FGD conducted after the campaign**

Those who were vaccinated said that they were informed about side-effects before they took OCV while others were not warned. Some who experienced side-effects after taking the vaccine said: “*You are not supposed to tell them the truth how it tastes*” to avoid panic among those not yet vaccinated. While those who did not experience side-effects dismissed such stories as lies, saying that these conditions were probably present before OCV uptake. Women in particular were identified as easily misdiagnosing side effects and of spreading rumors, or as one participant put it “*accusations towards the vaccine*” to “*other women*”.

### Access to the campaign

Table 4 shows that about half of both groups prefer access to the campaign on a Saturday while a third prefer Sunday, and about half of each group have a preference for mobile campaigns. Some among those travelling, sick, or at work during the announcement or the campaign period did not participate in OCV campaigns. Busy parents were not able to take the children for vaccination leading to the suggestion of using more time-consuming and door-to-door campaigns. Some who tried to access OCV found vaccination spots were out of doses and two unvaccinated participants reported that the volunteers can be “harsh” and unwelcoming based on past experiences. Others who participated fully reported taking advantage of the convenience of having the OCV delivered by the market place or door-to-door. Others who were “*feeling lazy to go*” for their vaccination were motivated by door-to-door mobilization. Others even took along visitors for vaccination out of fear that they could get cholera. Though most reported short or no queues, a few reported moderate efforts to access the vaccine, such as waiting in queues or going early to vaccination points. They indicated that more people would get vaccinated if OCV was part of routine care or available in their local clinic. Alternatively, OCV could be offered twice a month in the community or campaign periods should be extended. In either case, they thought that sensitization sessions should occur early in the morning before people leave for work. In the community, door-to-door delivery was suggested to mitigate barriers posed by distance or being physically challenged.

## Discussion

This is the first study on community perceptions on cholera in Zambia, which was necessary to understand uptake at this first introduction of OCV both as a reactive and preemptive vaccine in Zambia. Documenting reasons why some fully vaccinated while others did not vaccinate at all during the April and December 2016 campaigns in Lusaka, Zambia, provides critical information needed to prepare for more effective future campaigns. This information may also be used to shed light on the partial vaccination coverage found in Zambia particularly for regimens requiring more than one dose [33, 34].

In this study, those who took the two doses had a heightened awareness of conditions conducive to cholera and felt personally at risk of contracting cholera. They perceived OCV as necessary protection against cholera because they could not change their living conditions. This was similar to findings in a study conducted in the Democratic Republic of Congo by Sundaram et al. [35], where OCV was particularly favored by those who felt “trapped in an unclean environment” (p5). Conversely, as reported by Kumar et al. [36] and Peprah et al. [20] for other countries, people who perceived themselves as not susceptible to cholera due to good personal hygiene thought the vaccine unnecessary. Other reasons for low perceived susceptibility was adulthood and higher socio-economic status. However, those who felt helpless against the environment and thought that OCV did not reduce their risk of getting cholera, also chose not to be vaccinated. Thus, even though both groups saw cholera as life-threatening, those who did not vaccinate either thought of themselves as not susceptible or as helpless against their high susceptibility to cholera.

A greater proportion among those fully vaccinated (28% vs 15%) described cholera as being air-borne in the survey and in FGDs, as motivating them to take all possible precautions including OCV. Among those who did not vaccinate, the belief that OCV could only somewhat “prevent” but not “fully” protect against the “wind” guided their decision. Traditionally, “wind” is associated with the arrival of dangerous ghosts (*cibanda*) or the carrier of alien unidentified spirits (*imyela*) and therefore requiring supernatural intervention [37]. Illnesses attributable to the wind are common in West Africa, among Mali’s Senufo, where “kafélègè yama” (diseases of the wind) include measles, small pox, and eye infections [38]. Similar popular nosological entities (local classifications of diseases) are present among Guinea’s Soso [39] and Senegal’s Nyokholonke [40]. Further investigation is needed to understand whether in our study population, the biomedical definition of the concept “airborne” has been usurped [41, 42] or whether the belief that wind carries the spirits that enters the body maybe lending itself to the view that cholera is airborne.

Just as in other contexts, the fatalists vision of bodily permeability to diseases may reflect individual experiences of other forms of social victimization [42, 43]. Those who felt unable to change their environment and circumstances or ward off a curse felt that the vaccine could not protect them against the disease’s multiple entry points. Traditionally in Zambia, physical affliction would be considered as a manifestation of a social problem requiring a political solution [37] such as collective and government action to control cholera through improved wages and WASH as urged by our discussants. In this context, some legitimized their decision to not take the vaccine by suggesting that collective social action was more effective. They predict that those vaccinated will ultimately pay the price for relying on OCV when they caught cholera as the vaccine lost its potency in an environment harboring cholera.

Perceived benefits of taking OCV were mediated by the above perceptions of susceptibility and belief in the efficacy of OCV. As suggested by those who were vaccinated, the imminent danger posed by a cholera outbreak motivated uptake. However, in the absence of exposure, experience or information on OCV, those who did not vaccinate were influenced by rumours. Some were concerned that OCV was a method used by “white” people to eliminate them. Venturas & Umeh [44] reported a similar finding among health workers who predicted that the introduction of Human Papilloma Virus (HPV) vaccination in Zambia could give rise to allegations that Zambian recipients of HPV vaccines were being used as guinea pigs or being sterilized by westerners. A previous study suggests that distrust of health interventions in Zambia perhaps stems from a colonial history of usurpation expressed as concerns of exploitation and appropriation of bodies by people with power and/or knowledge [45].

Perceived benefits along with commonly understood etiology, disease classification and how remedies work on the sick body to restore health also mediated perceived risk. Some participants who suffered diarrhoea after taking OCV considered this expected side-effect as positive and as a sign of vaccine effectiveness. Green [46] found that Bantu groups (including the Bembas in Zambia) believed that dirt accumulating in the stomach can be the cause of several diseases (a popular nosological entity: “the snake in the belly”). In West Africa, dirt accumulation in the stomach needed purging [47]. In this configuration, the diarrhoea following uptake of a medicine (or vaccine) is considered as expulsion of that dirt.

In this study, reported side-effects of OCV such as bad taste, nausea, vomiting, rashes, and stomach pains, were unanticipated due to lack of complete information and sometimes interpreted as malevolence. The perception that something designed as “medicine” could in fact be “harmful” is rooted in the belief that witch doctors in Zambia used “mankwala wa chimuntu*”* (African medicine) not only to heal but to cause harm. This is similar to West Africa where the notion of “médicament” (medicine) covers comparable ambivalent notions [48] and is also historically reminiscent of the Greek “pharmakon*”* which can be a remedy, recipe, poison, philter [49]. This interpretation of malevolence, along with lack of statistics or experience such as that reported by our discussants, can increase skepticism about science and its products [41, 42]. Hence, as suggested for other settings, transparency and clear information on vaccine safety and efficacy is needed to address vaccine hesitancy in Lusaka [36, 50–52].

The recommendation for clear, accurate and targeted information given by some of our discussants appears sound. Quite a number of discussants acknowledged that symptoms maybe merely coincidental rather than a side-effect of OCV. However, without adequate information people were susceptible to rumors, traditionally attributed to malice among women in a polygamous society [37]. Thus, some discussants urged information on who should be vaccinated, the period of protection and side effects to counter the strong social influence on individual decision-making. Other practical suggestions included improving taste, size of bottle, and expanding campaigns to be on weekends and mobile to reach those who have competing commitments or are ill. The presence of wind related causation of cholera also suggests the need for regular communication using consistent messaging on cholera sources, prevention and treatment.

### Study limitations

We are unable to stratify our findings by the diverse ethnicity found in the compounds of urban Lusaka. We also cannot interpret our findings by length of stay in urban settings with presumably those more recently arrived from rural areas excluded from informational sources and steeped in tradition. Crucially, we had no means to verify self-reported vaccination status as the campaign only tabulated gender and age (child/adult) and did not distribute vaccination cards. Given the small number of discussions with those self-reported receiving both doses, we may have missed those completely opposed to OCV, who may still have fully vaccinated [53]. Nonetheless, our study produced rich data on complex decision-making by those living in crowded surroundings post-cholera outbreak. Using the framework of zero and two-dose OCV acceptors also provided the unique perspectives of the two groups across two campaigns 8 months apart. The use of qualitative methods provided the full spectrum of responses from those not and those fully vaccinated leading to this first time documentation of the association of cholera with “wind” and “air” in sub-Saharan Africa. Though mentioned by few in the short questionnaire, these terms were obsequious and the concept salient in the narratives.

As a qualitative study, our findings are not generalizable but maybe transferrable to other contexts and time. Any introduction of vaccines risks sub-optimal uptake. Our study shows that an, an operational qualitative inquiry can be carried out prior to introduction, even for reactive campaigns, to understand the emic (insider) views on susceptibility by gender, age, and other relevant stratification for the target area. Using open-ended questions, programme implementers can understand how the vaccine related disease is named, local theories of causation and prevention; perceived severity; who are the influencers and who should communicate on the vaccine, where and how should the vaccine be delivered.

## Conclusions

2017 marked the 200th anniversary of the first pandemic wave of cholera [54]. Much like the bacteria that has paradoxically thrived in modern times, vaccine hesitancy and skepticism of the science behind vaccines has risen in the age of information [55]. This skepticism must be addressed to meet Global Health’s goal of controlling emerging and re-emerging pathogens. In Lusaka compounds, vaccine hesitancy is a complex decision-making process rooted in circulating narratives, historical international interactions, and observable outcomes of vaccination. We recommend proactive transparency regarding sero-protection levels, delays and duration, possible side effects as well as experiences of past OCV campaigns conveyed by early and comprehensive communication to secure uptake. As the local explanatory model for both health and illness lies in the maintenance of social relationships, campaigns must emphasize collective uptake to achieve herd immunity as well as use social networks to provide accurate information on OCV.

## Appendix

**Table 5:** Health Belief Model Construct

HBM Construct	Major Data themes	0 Dose	2 Doses
Perceived Susceptibility	Cholera can strike anyone, anywhere being airborne and waterborne	+	++
	I can protect myself against cholera	++	
Perceived severity	Cholera is a killer disease	++	++
	Cholera is fast	++	++
Perceived benefits	Less sick persons after the first OCV round	+	++
	OCV seems safe	+	++
	There are absolutely no side effects		+
Perceived barriers	Cholera is air & water borne, can OCV work?	+	
	Vaccine is not serious, for children	+	
	No statistical evidence on OCV safety	+	
	There are mild side effects among vaccinated	++	
	Absent during campaign	++	
	Lack information on OCV to decide	+	
	The vaccination post was out of doses	+	
	The queues were long	++	+
	I avoid drugs in general	+	
	Taste is (said to be) bad	++	+
	Taste makes you want to vomit	++	+
	Overcome barrier: You get used to the taste		+
	Overcome barrier: Medicine is not supposed to taste good		+
Cues to action	Social influence (family, neighbors)	+	
	Requests Home visits, door to door campaign	+	
	Requests Longer campaigns (number of days)	+	+
Self-efficacy	I may be absent during the campaign hours	+	
	No need for OCV if following WASH recommendations.	+	

*+ Consistent and ++ very consistent with datum*

*0 D: Participants who took no OCV doses2D: Partcipants who took both OCV doses*

## Abbreviations

CIDRZ: Centre for Infectious Disease Research in Zambia
FGD: Focus Group Discussions
HPV: Human Papilloma Virus
KII: Key Informant Interviews
MoH: Ministry of Health
MSF: Médecins Sans Frontières (Doctors Without Borders)
NHRA: Zambian National Health Research Authority
OCV: Oral Cholera Vaccine
UNZABREC: University of Zambia Biomedical Research Ethics Committee
WASH: Water, sanitation, and hygiene
WHO: World Health Organization
ZMW: Zambian Kwacha
